# Hay Asthma

**Published:** 1846-02

**Authors:** 


*Hay Asthma.*—Dr. Swett also had met with another singular case: He had been called into the country to visit a gentleman 70 years of age, apparently laboring under asthma. The patient, who had always been healthy and free from cough, rose at night, laboring under a violent paroxysm of dyspnœa. A physician was called in, who bled him with some relief, but the disease recurred. The paroxysm would generally last 15 or 20 minutes, and then subsiding, the patient would remain free from distress for several hours. On one occasion, the interval had been as long as 48 hours. Dr. S. found the patient on his arrival apparently well, with the exception of slight oppression of breathing. On examination the respiration was found good, the action of the heart regular, and the pulse regular. Recollecting that asthma was occasionally brought on by breathing particular odors, Dr. S. inquired whether the patient had been exposed to anything unusual of the kind. The physician in attendance stated that he had, on the day of the attack, been employed in getting apples from a pit covered with hay. The hay and a portion of the apples had decayed, and gave out an offensive odor, which was probably the cause of the attack. Reference was then made to several cases, in which asthma had been produced by similar causes. The case of Dr. Perkins was mentioned, in whom asthma had been invariably produced by the smell of ipecacuanha. Dr. P. one day entered a drug store, and was instantly seized with the oppression of breathing, which precedes an attack. On inquiring, it was discovered that a clerk had two hours previously opened and exposed the powder of ipecac in weighing half an ounce. *Ibid.*

